# Iatrogenic Fistula in Hemodialysis Patients: An Alternative Approach to Thrombectomy of Arteriovenous Graft (AVG) Thrombosis

**DOI:** 10.1155/2022/2417980

**Published:** 2022-04-16

**Authors:** Ramanath Dukkipati, Alexandre M. Benjo, Antonio Jimenez, Ivo Lukitsch, Gift C. Echefu, Damodar R. Kumbala

**Affiliations:** ^1^Harbor-UCLA Medical Center, Torrance, California, USA; ^2^Lundquist Research Institute, Torrance, California, USA; ^3^UCLA School of Medicine, Westwood, California, USA; ^4^Lake Side Heart and Vascular Center, Lake Havasu, Arizona, USA; ^5^Puerto Rico Vascular Center, Puerto Rico, USA; ^6^Division of Nephrology, Ochsner Medical Center, New Orleans, Louisiana, USA; ^7^Baton Rouge General Medical Center, Internal Medicine Program, Baton Rouge, Louisiana, USA; ^8^Renal Associates of Baton Rouge, Baton Rouge, Louisiana, USA

## Abstract

Arterial venous (AV) fistula is the first choice of vascular access to perform hemodialysis in the vast majority of suitable patients followed by arteriovenous grafts (AVG). An iatrogenic fistula can occur when a second vein adjacent to the graft is punctured and the needle traverses the vein. In normal circumstances, this has no clinical repercussions and does not need correction, and in prior reports, it has helped to maintain the patency of partially occluded grafts but rarely can lead to thrombosis of the graft due to reduced flow and pressure in the graft lumen. We report here what we believe is a unique approach to perform thrombectomy of an occluded graft in a 71-year-old patient on hemodialysis to avoid placement of tunneled hemodialysis catheters and complications associated with catheters. When the outflow of basilic vein in this patient was thrombosed and could not be traversed, we successfully used an iatrogenic fistula as main outflow vein for the graft and created an alternative vein for drainage thus avoiding placement of a tunneled catheter for hemodialysis.

## 1. Introduction

The basilic vein is the larger vein in diameter compared to the cephalic vein. It is a commonly used vein for creation of arteriovenous fistula (AVF) in the upper extremity [1]. Basilic vein is also used as the outflow vein for arteriovenous grafts (AVG). Cephalic arch stenosis and thrombosis are a complication seen in patients with brachial artery-cephalic vein fistulas [2]. Thrombosis of these two upper extremity fistulas requires urgent endovascular intervention to salvage them to avoid the associated complications associated with tunneled hemodialysis catheters in the event thrombectomy of the fistula is unsuccessful. According to the 2019 KDOQI guidelines, the selection of a specific hemodialysis access is an individualized shared decision-making process aimed at providing the right access for the right patient, with a focus on vessel preservation, access creation, contingency, and succession plans [3]. Arteriovenous fistula is considered the first line choice for permanent vascular access in hemodialysis (HD) patients while AV graft is a second line option [4]. Innovative approaches for salvage of thrombosed AVF's to extend their life span are urgently needed. We present an innovative endovascular approach to salvage a thrombosed basilic vein fistula that extended the use of this fistula to receive HD for our patient. Salvage of thrombosed AVF or AVG avoids complications related to tunneled dialysis catheters.

## 2. Case Presentation

We present a 71-year-old male with ESRD on hemodialysis for 6 months who was deemed not suitable for creation of a fistula due to his suboptimal vein size and therefore had surgical creation of right brachial-basilica AV graft. Eight months after placement of the AVG, he presented with complete loss of thrill over the AVG. A few weeks prior to that adequacy of hemodialysis access was declining during the InterAccess flow monitoring which was below 500 ml/min. Patient was referred for a fistulogram but was unable to follow up prior to thrombosis of access. An ultrasound duplex scan demonstrated thrombosis in the AVG and therefore patient was referred to us for declotting.

**Figure 1:**
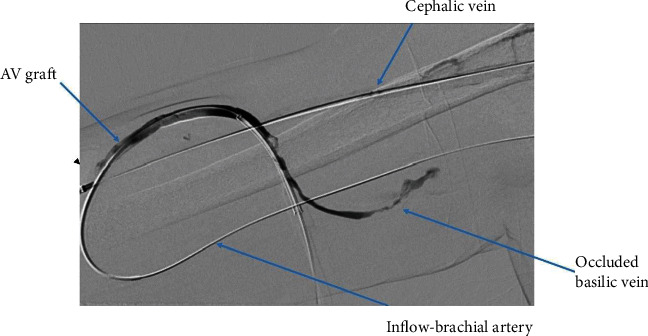
Thrombosed basilic vein.

**Figure 2:**
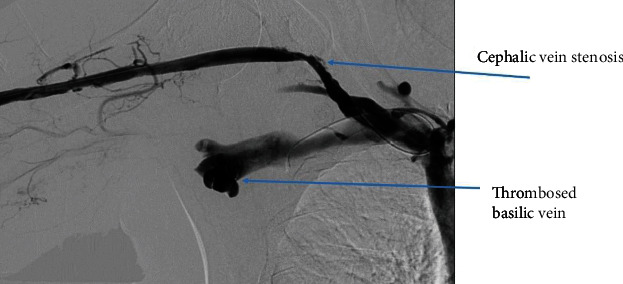
Outflow obstruction—cephalic vein stenosis and basilic vein thrombosis.

**Figure 3:**
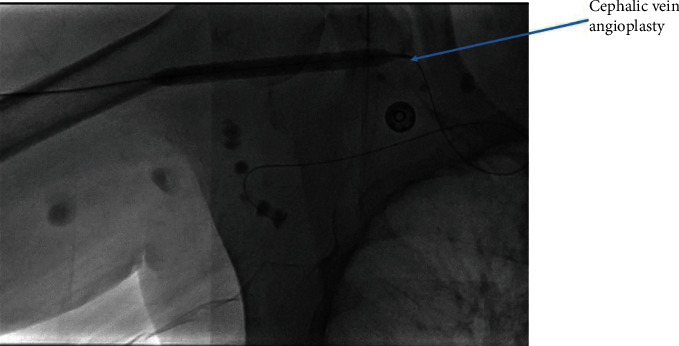
Outflow angioplasty.

**Figure 4:**
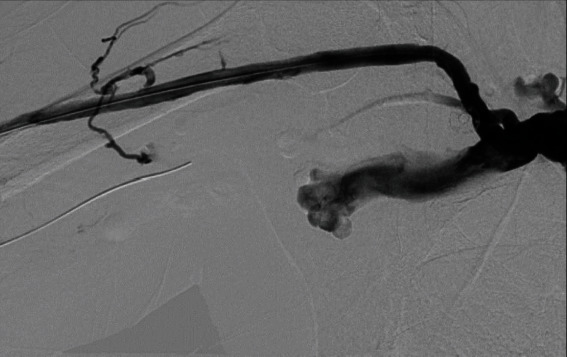
Outflow final result.

**Figure 5:**
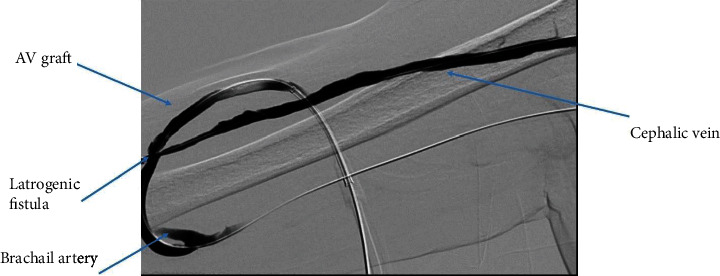
Post cephalic vein thrombectomy/percutaneous angioplasty.

**Figure 6:**
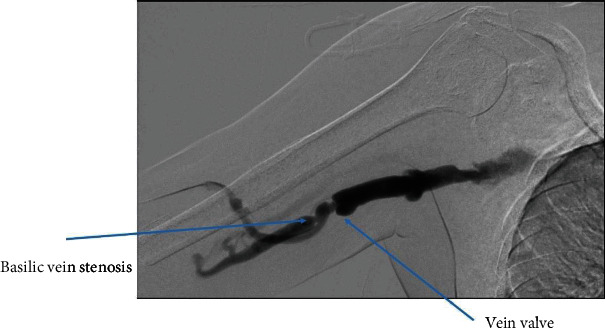
Relook after 2 months—self recanalization of basilic vein with significant stenosis.

**Figure 7:**
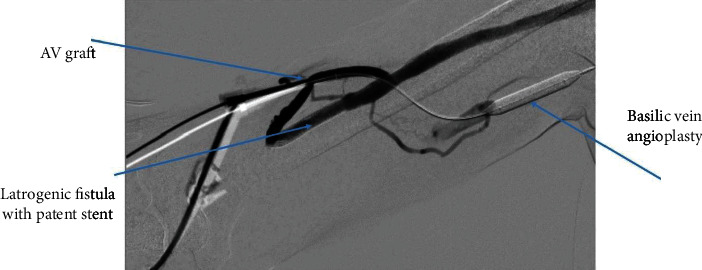
Patent graft to cephalic vein stent and basilic vein angioplasty.

**Figure 8:**
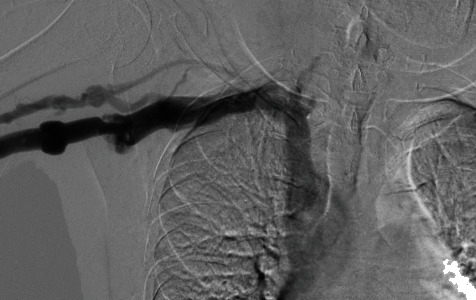
Basilic vein postangioplasty.

We cannulated the graft in antegrade direction. Angiogram showed that the basilic vein was thrombosed (Figure 1). We could not cross the basilic vein occlusion in both antegrade and retrograde direction with soft and stiff 0.18 and 0.35 mm wires, and, therefore, we opted to attempt thrombectomy of the cephalic vein and outflow. Initially, we passed a 0.35 mm soft and angled hydrophilic (Terumo) wire in to the outflow performing rheolytic aspiration followed by balloon maceration of the remaining thrombus burden. We also noted a completely thrombosed basilic vein (Figure 1) during the first injection of radiocontrast and a partially clotted cephalic vein which was draining from the graft that drained the graft flow (Figure 2). Angioplasty of the cephalic arch stenosis was performed (Figures 3 and 4). A severe outflow lesion with subclavian vein stenosis was also observed. Additionally, we performed balloon angioplasty of the subclavian vein stenosis which was followed by declotting the arterial limb with a 5.5 French size Fogarty catheter. We were able to establish optimal flow with excellent back flow, normal thrill, and absence of pulsations. To ensure patency of this iatrogenic fistula, we placed a covered stent 7 mm × 5 cm stent (Viabahn) at the site where the graft drains into the cephalic vein, and angiogram showed good flow from the graft into the cephalic vein (Figure 5). Postoperatively, he underwent successful hemodialysis through access. We electively brought the patient two months later for readdressing the basilic vein thrombosis as the clot burden was much larger than the clot seen in the cephalic vein (Figure 6). Once the outflow was established for the graft through the cephalic vein with creation of the iatrogenic fistula, we observed spontaneous resolution of the basilic vein thrombosis and we did angioplasty of the basilic vein (Figures 7 and 8 ). Angiogram after the angioplasty of the basilic vein showed excellent flow in the basilic vein all the way into the central veins (Figure 8). The patient had successful cannulation of the graftula (one needle in the graft and the other in the vein) for hemodialysis on the next day using the graft segment for arterial needle placement and the stent area for venous needle placement. He has received hemodialysis without any problems with optimal blood flow or cannulation problems for several months following our intervention.

## 3. Discussion

An iatrogenic fistula is a conduit created between the arteriovenous graft and nearby vein(s), described as a consequence of repetitive traumatic cannulation of the AV graft, which leads to establishment of an alternative pathway of venous outflow of the graft. The repeated cannulations can lead to a hematoma in the surrounding tissue. This results in creation of a fistula with near native veins and can remain asymptomatic and nonfunctioning. Thrombosis of the graft leads to increased intragraft pressure in the venous arm of the AV graft which leads to increased blood flow through the iatrogenic fistula [5, 6]. The iatrogenic fistula could lead to AV graft dysfunction and decreased blood flow through the graft and may lead to thrombosis of the venous arm of the graft [6]. Most cases of iatrogenic fistulas do not require an intervention but with appropriate management at the correct time they resolve on their own. In this unique case, we were unsuccessful in establishing the patency of the basilic vein (the main outflow of the AV graft in this patient) due to severe stenosis and large clot burden, and, therefore, we used iatrogenic fistula as an alternative outflow vein. A stent was placed where the graft is draining to the cephalic vein to maintain patency as vein-graft interface segment stenosis is the leading cause of graft thrombosis. This resolution of outflow obstruction made it possible for this patient to continue his dialysis without the need for placement of a tunneled dialysis catheter. This prevented our patient from the complications associated with placement of vascular catheters for hemodialysis. This intervention allowed adequate time to lapse for spontaneous resolution of the basilic vein thrombosis.

Common complications of fistulas and grafts in hemodialysis patients include thrombosis, infection, steal syndrome, pseudoaneurysms with risk of bleeding, venous hypertension, seromas, and heart failure. Thrombosis and infection occur more frequently with grafts than with fistulas but the patency advantage of fistulas over grafts is not found in people who are 65 years and older [7–9]. Thrombosis of AV grafts in HD patients is most often due to stenosis at the anastomosis of the graft to vein and is one of the most common cause of vascular access malfunction, typically in the range of 0.6 to 1.2 per patient-year [10–12]. The goal of chronic vascular access is to offer repetitive access to blood circulation with minimal complications. Difficulties in vascular access generate significant problems to the healthcare providers, the healthcare system, and especially the patients with end-stage renal disease where a failing access is the leading cause of hospitalizations for patients on HD. An autogenous AV fistula using the patient's native tissues is still the preferred form of permanent vascular access, but when creation of a fistula is not possible, a graft should be considered [13]. Greater than 80 percent of thrombosed grafts have a stenotic lesion and often the site of lesion is in the anastomosis segment of the outflow vein to the graft. Risk factors for graft thrombosis are presence of stenotic lesion, infection, pseudoaneurysm, hypotension, hypovolemia, excessive compression, hypercoagulable state, and trauma [14]. Potential complications during percutaneous thrombectomy include pulmonary emboli, distal arterial embolization, and rupture of the AV access.

In our case, the cause of thrombosis we suspect could be that the graft-to-vein fistula reducing intraluminal pressure in the graft played a key role in causing graft thrombosis [15]. We suspect that the fistula formed after repeated inadvertent concomitant cannulations of the vascular graft and the adjacent cephalic vein during hemodialysis. In the presence of other risk factors, the graft–to-vein fistula can predispose to graft thrombosis by steal phenomenon. Consistent with the scenario, especially in the presence of venous stenosis at the anastomosis site, significant amount of blood steal through the iatrogenic fistula can decrease blood flow in the venous limb of graft and may leaded to thrombosis.

The iatrogenic fistula formation between AV graft and an adjacent vein is a rare but a reported cause of graft thrombosis. The prevalence of this complication is not well established, but the major contributing factor is repeated inadvertent concomitant cannulation of AV graft and adjacent vein in the presence of venous outflow stenosis. Careful selection of cannulation site and proper cannulation technique is extremely important to prevent this rare complication. Early detection and resolution of venous outflow stenosis are of high importance in preventing and treating this complication. These iatrogenic fistulas only manifest during periods of elevated graft pressure. Once the graft pressure is normalized, these fistulas have minimal hemodynamic effect, and some resolve spontaneously without the need of any specific treatment [16].

In conclusion, although a traumatic graft-to-vein fistula is not a common hemodialysis access complication to occur, it can lead to graft thrombosis. Careful cannulation technique is important to prevent the development of iatrogenic fistulas. Endovascular techniques can be utilized to salvage this type of thrombotic traumatic AV fistulas using different outflow vein than the original vein the graft was anastomosed to.

## Data Availability

The data presented in this study are available on request from the corresponding author. The data are not publicly available due to ethical and privacy.
